# Performance of breast cancer risk prediction algorithms across mammography systems in the UK screening programme

**DOI:** 10.1038/s41746-026-02507-7

**Published:** 2026-03-08

**Authors:** Joshua Rothwell, Nicholas Payne, Fleur Kilburn-Toppin, Yuan Huang, Joshua Kaggie, Richard Black, Sarah Hickman, Bahman Kasmai, Arne Juette, Fiona Gilbert

**Affiliations:** 1 University of Cambridge, Department of Radiology, Cambridge, UK; 2 Cambridge University Hospitals NHS Foundation Trust, Department of Radiology, Cambridge, UK; 3 University of Cambridge, EPSRC Cambridge Mathematics of Information in Healthcare Hub, Cambridge, UK; 4 Barts Health NHS Trust, Department of Radiology, London, UK; 5 Norfolk and Norwich University Hospital, Department of Radiology, Norwich, UK

**Keywords:** Cancer, Computational biology and bioinformatics, Oncology

## Abstract

Thirty percent of interval breast cancers, diagnosed between routine screening mammograms, have a poorer prognosis than screen-detected cancers. Deep learning algorithms can estimate short-term risk from negative mammograms to guide supplemental imaging or screening intervals, but comparative validation on complete national screening data is lacking. We retrospectively evaluated four risk algorithms (Mirai, iCAD, Transpara, and Google) using 112,621 negative mammograms from two UK NHS Breast Screening Programme sites with different mammography systems (Philips, GE) over one screening round (2014–2017) with five-year follow-up, including 1225 future cancers. There was a distinct ranking in discriminative ability; overall AUCs ranged 0.65–0.72, only one algorithm significantly differed between systems. For interval cancers, AUCs ranged 0.67–0.77. Within the highest 4.0% of risk scores, top algorithms identified ~20% of future cancers, including ~27% of interval cancers, doubling at the 14.0% threshold. These differences highlight the need for multi-algorithm prospective trials and potential fine-tuning to improve generalisation across unseen systems.

## Introduction

Early detection of breast cancer with mammography screening programmes has been shown to decrease breast cancer mortality^1^. However, it is estimated that thirty percent of breast cancers that present between routine screening examinations are associated with more advanced stages at diagnosis and poorer prognoses than screen-detected cancers^2–5^. Risk of these ‘interval’ cancers (ICs) increases with longer screening intervals – 12–24 months after negative screening, incidence was found to be double that of the first 0-12 months in eight European programmes (Törnberg et al.)^6^ and the NHS Breast Screening Programme (NHSBSP) (Bennett et al.)^7^.

Personalised screening utilizes questionnaires and polygenic risk scores to estimate lifetime breast cancer risk for women. However, deep learning (DL) algorithms can use features present within negative screening mammograms to predict short-term breast cancer risk, identifying women who may benefit from more frequent screening^8,9^ or more sensitive imaging modalities, such as MRI or contrast-enhanced mammography (CEM)^10,11^. Such algorithms have been retrospectively validated^12–15^ and found to outperform models that account for traditional risk factors but do not incorporate mammographic features^16,17^. Furthermore, Hill et al. suggest that using DL risk scores to personalise NHSBSP screening intervals may be cost-effective with appropriate operating threshold selection^18^.

Ultrasound is advocated as supplemental imaging in some countries for women with dense breasts^19^. However, the DENSE trial showed increased sensitivity using MRI^20^ and interim results of the BRAID study confirmed that contrast techniques, such as abbreviated MRI or CEM, are equally effective^11^. However, further research is needed to optimize the selection of women for additional imaging, beyond breast density alone. Interim results of the ScreenTrustMRI trial demonstrated the value of using DL risk scores (incorporating mammography-derived risk, subjective reading difficulty, and cancer signs) to select women for MRI, with a cancer detection rate of 64.0 per 1,000 examinations^21^.

The objective of this study was to retrospectively compare state-of-the-art available DL risk prediction algorithms using national NHSBSP data from two different screening sites, which used different digital mammography systems. The primary objective was to determine algorithm performance characteristics, to contextualise and benchmark algorithms against previously published results. The secondary objective was to evaluate algorithm predictions at clinically meaningful operating thresholds, to quantify potential clinical impact and inform future implementation strategies^22^.

## Results

### Study cohort

**Fig. 1:**
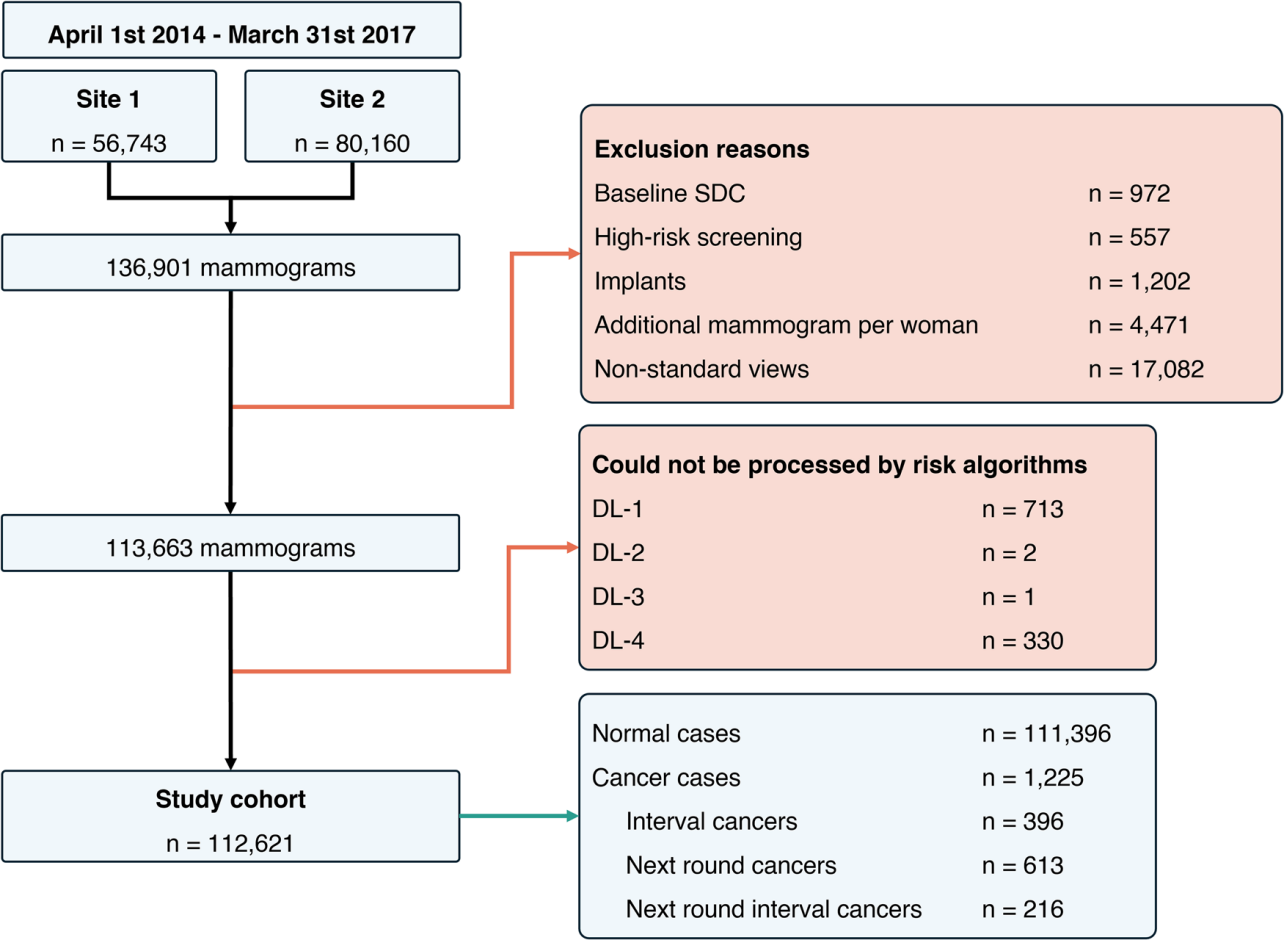
Study cohort selection. Flowchart demonstrating exclusion of mammograms from the study cohort.

Of 136,901 mammograms, 17.0% (*n* = 23,238) were excluded, with reasons including non-standard views (*n* = 17,082), additional mammograms per woman (*n* = 4471), and implant DICOM tags (*n* = 1202) (Fig. 1). The median excluded age was 59-years (IQR: 53, 66). For the dataset (*n* = 113,663), initial processing failure rates were 0.99% (*n* = 1121), 0.01% (*n* = 16), 0.00% (*n* = 2), and 0.30% (*n* = 337) of mammograms for DL-1-to-4, respectively. A systematic algorithm failure analysis was undertaken, and successfully reprocessed mammograms were included if all algorithm scores became available. Failure numbers and required processing corrections (including modification or removal of specific DICOM tags, and age-range exclusions) are described in the Supplementary Information (Note 1). Processing times varied substantially between algorithms and vendors (Table S1). However, median time per examination ranged 8–79 seconds, indicating suitability for batch processing and potential feasibility for real-time use, particularly for faster algorithms (DL-1-and-3). All algorithms took longer to process Philips mammograms compared with GE, likely due to the larger image size.

**Fig. 2:**
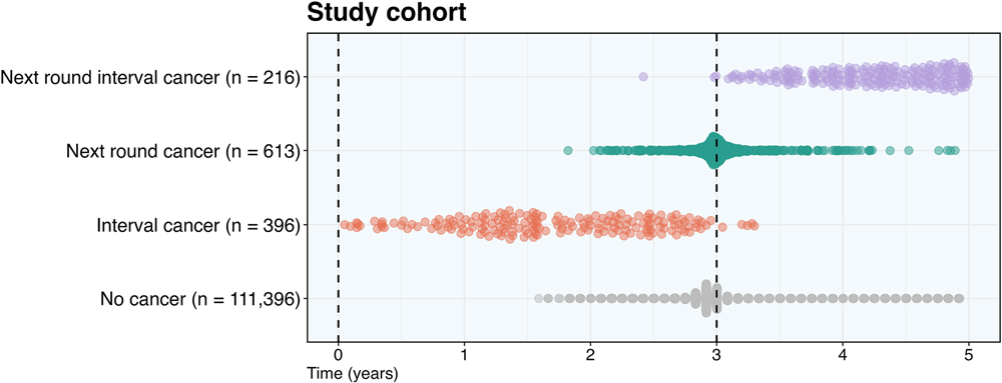
Time to diagnosis for future cancers and screening round length for normal cases. Overview of the time-to-diagnosis for all future cancers and the screening round length for normal (no cancer) cases included within the study cohort. The time of the baseline screening mammogram (0 years) and the NHS Breast Screening Programme target subsequent screening appointment (36-months) are both represented by black dashed lines. Screening dates for normal cases were pseudonymised to the first day of the month, so appear quantised.

Risk scores were ultimately obtained and analysed for 112,621 negative screening mammograms (median age: 59-years, IQR: 52, 65), including 1,225 future cancers – 396 interval cancers (ICs), 613 next-round screen-detected cancers (NRCs), and 216 next-round interval cancers (NRICs). 44.3% (*n* = 49,938) were obtained from Philips mammography systems, and 55.7% (*n* = 62,683) on GE systems (Table 1). Following baseline screening, the median time to cancer diagnosis was 586 days (IQR: 425, 871) for ICs, 1092 days (IQR: 1067, 1127) for NRCs, and 1566 days (IQR: 1398, 1715) for NRICs. Median screening round length was 1,065 days (IQR: 1064, 1096) for normal cases (Fig. 2). Further characteristics are reported in Table 1.

**Table 1:** Study cohort characteristics

	All examinations	Normal cases	Future cancers			
			All	ICs only	NRCs only	NRCs only
**Total number**	112,621	111,396	1,225	396	613	216
**Per 1000 women**	..	..	10.9	3.5	5.4	1.9
**Patient age (years)**						
**Median [IQR]**	59 [52,65]	59 [52,65]	59 [53,65]	59 [53,66]	60 [54,65]	57 [51,65]
**≤54**	33.9% (38,164)	33.9% (37,790)	30.5% (374)	32.8% (130)	25.3% (155)	41.2% (89)
**55-59**	19.2% (21,589)	19.2% (21,346)	19.8% (243)	18.7% (74)	21.0% (129)	18.5% (40)
**60-64**	17.2% (19,418)	17.2% (19,182)	19.3% (236)	17.2% (68)	22.3% (137)	14.4% (31)
**≥65**	29.7% (33,450)	29.7% (33,078)	30.4% (372)	31.3% (124)	31.3% (192)	25.9% (56)
**Mammography system**						
**Philips**	44.3% (49,938)	44.3% (49,382)	45.4% (556)	43.9% (174)	46.8% (287)	44.0% (95)
**GE**	55.7% (62,683)	55.7% (62,014)	54.6% (669)	56.1% (222)	53.2% (326)	56.0% (121)
**Cancer grade**						
**I**	..	..	15.0% (184)	9.6% (38)	20.6% (126)	9.3% (20)
**II**	..	..	40.2% (493)	40.9% (162)	39.5% (242)	41.2% (89)
**III**	..	..	22.4% (275)	29.0% (115)	15.3% (94)	30.6% (66)
**DCIS**	..	..	14.4% (177)	8.8% (35)	19.6% (120)	10.2% (22)
**Unavailable**			7.8% (96)	11.6% (46)	5.1% (31)	8.8% (19)
**Molecular subtype**						
**Luminal A (ER** + **/PR** + **/HR2-)**	..	..	20.7% (253)	21.5% (85)	25.8% (158)	4.6% (10)
**Luminal B (ER** + **/PR-/HR2-)**	..	..	5.0% (61)	5.3% (21)	5.1% (31)	4.2% (9)
**Luminal B (ER** + **/PR** + **/-/HR2** + **)**			3.0% (37)	3.0% (12)	3.6% (22)	1.4% (3)
**HER2 positive (ER-/PR-/HR2** + **)**	..	..	1.9% (23)	1.8% (7)	1.5% (9)	3.2% (7)
**Triple negative (ER-/PR-/HR2-)**	..	..	3.0% (37)	3.8% (15)	1.8% (11)	5.1% (11)
**Unavailable**	..	..	66.4% (814)	64.6% (256)	62.3% (382)	81.5% (176)

Median age is accompanied by the interquartile range in brackets. Percentages are accompanied by the number of corresponding mammograms in parentheses. Molecular subtype was only available for one site. *ICs* Interval Cancers, *NRCs* Next-Round screen-detected Cancers, *NRICs* Next-Round Interval Cancers.

### Overall performance

**Fig. 3:**
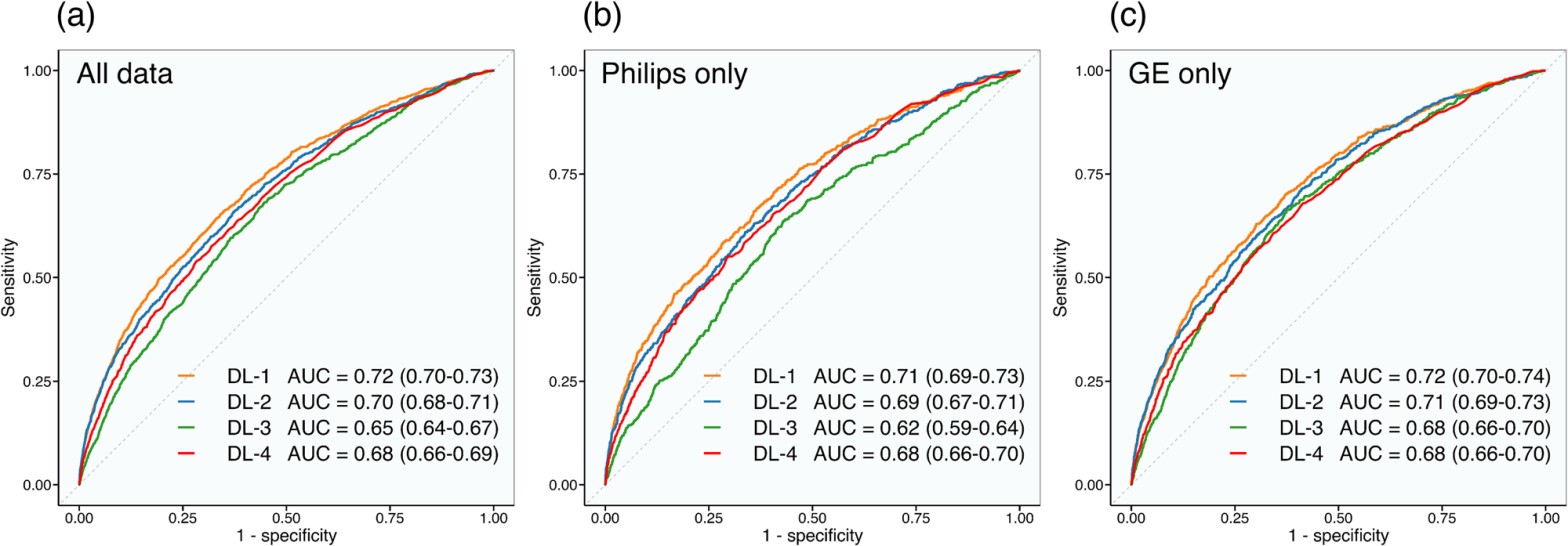
Algorithms receiver operating characteristic curves for all data and individual mammography system vendors. Receiver operating characteristic curves and corresponding AUCs for each risk prediction algorithm using all data (**a**), mammograms obtained on Philips mammography systems only (**b**), and mammograms obtained on GE mammography systems only (**c**). DL Deep Learning algorithm. AUC Area Under the receiver operating characteristic Curve.

The areas under the receiver operating characteristic curves (AUCs) were 0.72 (95% CI 0.70–0.73), 0.70 (95% CI 0.68–0.71), 0.65 (95% CI 0.64–0.67), and 0.68 (95% CI 0.66–0.69), for DL-1-to-4, respectively (Fig. 3a). Adjusted pairwise comparisons confirmed significant differences mirroring the AUC order; DL-1 performed best, and DL-3 performed the lowest (all *p* < 0.001; except DL-1-vs-2: *p* = 0.004, and DL-3-vs-4: *p* = 0.006).

Reported in sequential order (DL-1-to-4), AUCs for Philips mammograms alone were 0.71 (95% CI 0.69–0.73), 0.69 (95% CI 0.67–0.71), 0.62 (95% CI 0.59–0.64), and 0.68 (95% CI 0.66–0.70) (Fig. 3: (b)). DL‑1 significantly outperformed all other algorithms (DL-2: *p* = 0.050; DL-3: *p* < 0.001; DL-4: *p* = 0.002); DL-2-and-4 were statistically comparable (*p* = 0.25) and both outperformed DL-3 (*p* < 0.001). AUCs for GE mammograms were 0.72 (95% CI 0.70–0.74), 0.71 (95% CI 0.69–0.73), 0.68 (95% CI 0.66–0.70), and 0.68 (95% CI 0.66–0.70) (Fig. 3: (c)). DL-1-and-2 were comparable (*p* = 0.19) and both outperformed DL-3-and-4 (*p* ≤ 0.002), which were comparable (*p* = 0.84). All AUCs were statistically comparable between systems except DL-3, which was lower for Philips (0.62 [95% CI 0.59–0.64]) than GE (0.69 [95% CI 0.67–0.71]; *p* < 0.001). Calibration analyses stratified by mammography vendor are provided in the Supplementary Information (Note 2).

### Future cancer subgroups

**Fig. 4:**
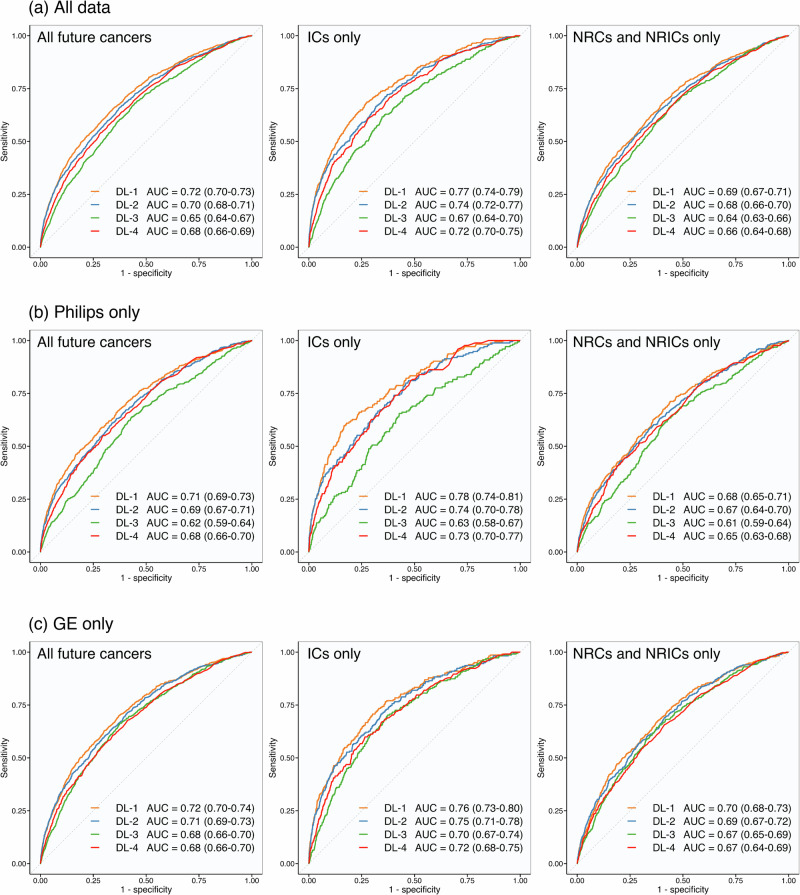
Algorithms receiver operating characteristic curves for different future cancer types. Receiver operating characteristic curves and corresponding AUCs for each risk prediction algorithm across all data and two subgroups: normal cases with ‘earlier’ future cancers (ICs only), and normal cases with ‘later’ future cancers (NRCs and NRICs only). Results are provided for all data (**a**), mammograms obtained on Philips mammography systems only (**b**), and mammograms obtained on GE mammography systems only (**c**). DL Deep Learning algorithm, IC Interval Cancer, NRCs Next-Round screen-detected Cancers, NRICs Next-Round Interval Cancers.

DL-1 was best at discriminating between normal cases and ‘early’ future cancers (ICs alone), with a higher AUC (0.77 [95% CI 0.74–0.79]) than all other algorithms (DL-3-and-4: *p* < 0.001; DL-2: *p* = 0.007) (Fig. 4a). DL-2 (0.74 [95% CI 0.72–0.77]) and DL-4 (0.72 [95% CI 0.70–0.75]) were statistically comparable (*p* = 0.078), and both outperformed DL-3 (0.67 [95% CI 0.64–0.70]; *p* < 0.001).

In comparison, discriminating between normal cases and ‘later’ future cancers (NRCs and NRICs alone), significant decreases were found for DL-1 (0.77–0.69 [95% CI 0.67–0.71]; *p* < 0.001), DL-2 (0.74 to 0.68 [95% CI 0.66–0.70]; *p* = 0.004), and DL-4 (0.72 to 0.66 [95% CI 0.64–0.68]; *p* < 0.001), but not DL-3 (0.67–0.65 [95% CI 0.63–0.66]; *p* = 0.101). Within this ‘later’ future cancer subgroup, DL-1’s remained highest (0.70), but were comparable with DL-2 (0.69; *p* = 0.14); both outperformed DL-3 (0.65; *p* < 0.001). DL-1 outperformed DL-4 (0.66; *p* < 0.001), but DL-2 was comparable (*p* = 0.097), with no difference between DL-3-and-4 (*p* = 0.14).

Restricted to Philips mammograms (Fig. 4b), AUC patterns were consistent with the overall data. Discriminating between normal cases and ‘early’ future cancers, DL-1 (0.78) outperformed DL-2 (0.74; *p* = 0.02), DL-3 (0.62; *p* < 0.001), and DL-4 (0.73; *p* = 0.02). Again, DL-2-and-4 were comparable (*p* = 0.77) and both outperformed DL-3 (*p* < 0.001). Compared to the ‘later’ future cancers subgroup, patterns remained consistent: decreases were found for DL-1 (0.78 to 0.68; *p* < 0.001), DL-2 (0.74 to 0.67; *p* = 0.003), and DL-4 (0.73 to 0.65; *p* < 0.001), but not DL-3 (0.63 to 0.61; *p* = 0.65). Within this subgroup, DL-1’s AUC remained highest (0.69), did not differ from DL-2 (0.67; *p* = 0.47) and trended against DL-4 (0.66; *p* = 0.058). All outperformed DL-3 (0.61; DL-1-and-2: *p* < 0.001; DL-4: *p* = 0.026).

Restricted to GE mammograms (Fig. 4: (c)), performance differences were smaller. Discriminating between normal cases and ‘early’ future cancers, DL-1 (0.76) was superior to DL-3 (0.70; *p* < 0.001) and DL-4 (0.72; *p* = 0.002), but comparable with DL-2 (0.75; *p* = 0.36), with no other pairwise IC comparisons significant. Compared to the ‘later’ future cancers subgroup, decreases were seen for DL-1 (0.76 to 0.70; *p* = 0.004), DL-2 (0.75 to 0.70; *p* = 0.011), and DL-4 (0.72 to 0.67; *p* = 0.016), but not DL-3 (0.70 to 0.67; *p* = 0.12). Within this subgroup, DL-1 (0.70) and DL-2 (0.70) were comparable (*p* = 0.54); both outperformed DL-3 (0.67; DL-1: *p* = 0.011; DL-2: *p* = 0.079) and DL-4 (0.67; DL-1: *p* = 0.003; DL-2: *p* = 0.044). DL-3 and DL-4 were comparable (*p* = 0.63). Unabridged results with confidence intervals are provided in the Supplementary Information (Note 3).

### Future cancer predictions at clinically relevant operating thresholds

Within the highest 4.0% of their respective risk scores, 19.7%, 19.3%, 10.4%, and 14.9% of all future cancers (n = 1,225) were correctly predicted by DL-1-to-4. In the corresponding sequential DL order, this included 27.5%, 25.8%, 12.1%, and 18.7% of all ICs (*n* = 396), 16.5%, 18.4%, 10.1%, and 13.7% of all NRCs (*n* = 613), and 14.4%, 9.7%, 8.3%, and 11.1% of all NRICs (*n* = 216). Cochran’s Q tests found heterogeneity between all future cancer proportions, ICs alone, and NRCs alone (all *p* < 0.001), but not for NRICs alone (*p* = 0.081). Post-hoc pairwise McNemar comparisons are reported as follows: proportions of correctly predicted future cancers for DL-1-and-2 were comparable (*p* = 0.76); both were significantly higher than DL-3-and-4 (all *p* < 0.001), with DL-4 surpassing DL-3 (*p* < 0.001). The same pattern held for ICs only: DL-1-and-2 were comparable (*p* = 0.45); both exceeded DL-3-and-4 (*p* ≤ 0.003), and DL-4 was higher than DL-3 (*p* = 0.009). For NRCs alone, DL-1-and-2 were comparable (*p* = 0.27); DL-2 outperformed DL-3-and-4 (*p* ≤ 0.004); DL-1 exceeded DL-3 (*p* < 0.001) but not DL-4 (*p* = 0.11); DL-3-and-4 were comparable (*p* = 0.11).

Using logistic regression to evaluate the subset for which Volpara density grade was available, true positive cancer predictions were more likely in dense breasts for DL-1 (*p* = 0.013), and in older women for DL-2 (*p* = 0.012), DL-3 (*p* = 0.009), and DL-4 (*p* = 0.014). Pairwise McNemar’s tests found no statistical differences between the proportions of correctly predicted cancer characteristics following adjustment (Table 3).

In comparison, within the highest 14.0% of their respective risk scores, 41.8%, 38.0%, 30.4%, and 35.7% of all future cancers were correctly predicted by DL-1-to-4. In the corresponding sequential DL order, this included 50.3%, 45.5%, 32.6%, and 41.9% of all ICs, 39.3%, 37.8%, 31.0% and 32.8% of all NRCs, and 33.3%, 25.0%, 24.5%, and 32.4% of all NRICs. Cochran’s Q tests again found heterogeneity in the proportions of correctly predicted future cancers, ICs alone, and NRCs alone (all *p* < 0.001), but also for NRICs alone (*p* = 0.004). However, follow-up pairwise McNemar’s tests did not identify any significant differences between individual algorithm NRIC proportions following adjustment. For other pairwise comparisons: the proportion of future cancers correctly predicted by DL-1 was higher than DL-2 (*p* = 0.009) and DL-4 (*p* < 0.001); DL-2-and-4 were comparable (*p* = 0.062), and all outperformed DL-3 (*p* ≤ 0.001). For ICs alone, DL-1-and-2 were comparable (*p* = 0.11); DL-1 exceeded DL-4 (*p* = 0.002), DL-2-and-4 were comparable (*p* = 0.16), and all outperformed DL-3 (*p* < 0.001). For NRCs alone, DL-1-and-2 were comparable (*p* = 0.93) and both higher than DL-3-and-4 (DL-1: *p* < 0.001; DL-2: *p* ≤ 0.007); DL-3-and-4 were comparable (*p* = 0.93).

Using logistic regression to exclusively evaluate the subset for which Volpara density grade was available, correctly predicted cancers were still more likely to be in dense breasts for DL-1 (*p* < 0.001) and DL-4 (*p* = 0.043), and in older women for DL-1 (*p* = 0.011), DL-2-and-3 (both *p* < 0.001), and DL-4 (*p* = 0.013). Adjusted pairwise McNemar’s tests found DL-1-and-2 to both predict more future cancers than DL-3 (*p* ≤ 0.003), as well as more grade II cancers specifically (*p* ≤ 0.004). In the dense-breast subgroup, DL-1 found more cancers than DL-2-and-3 (*p* ≤ 0.007), and DL-4 more than DL-3 (*p* = 0.038) (Table 4).

**Fig. 5:**
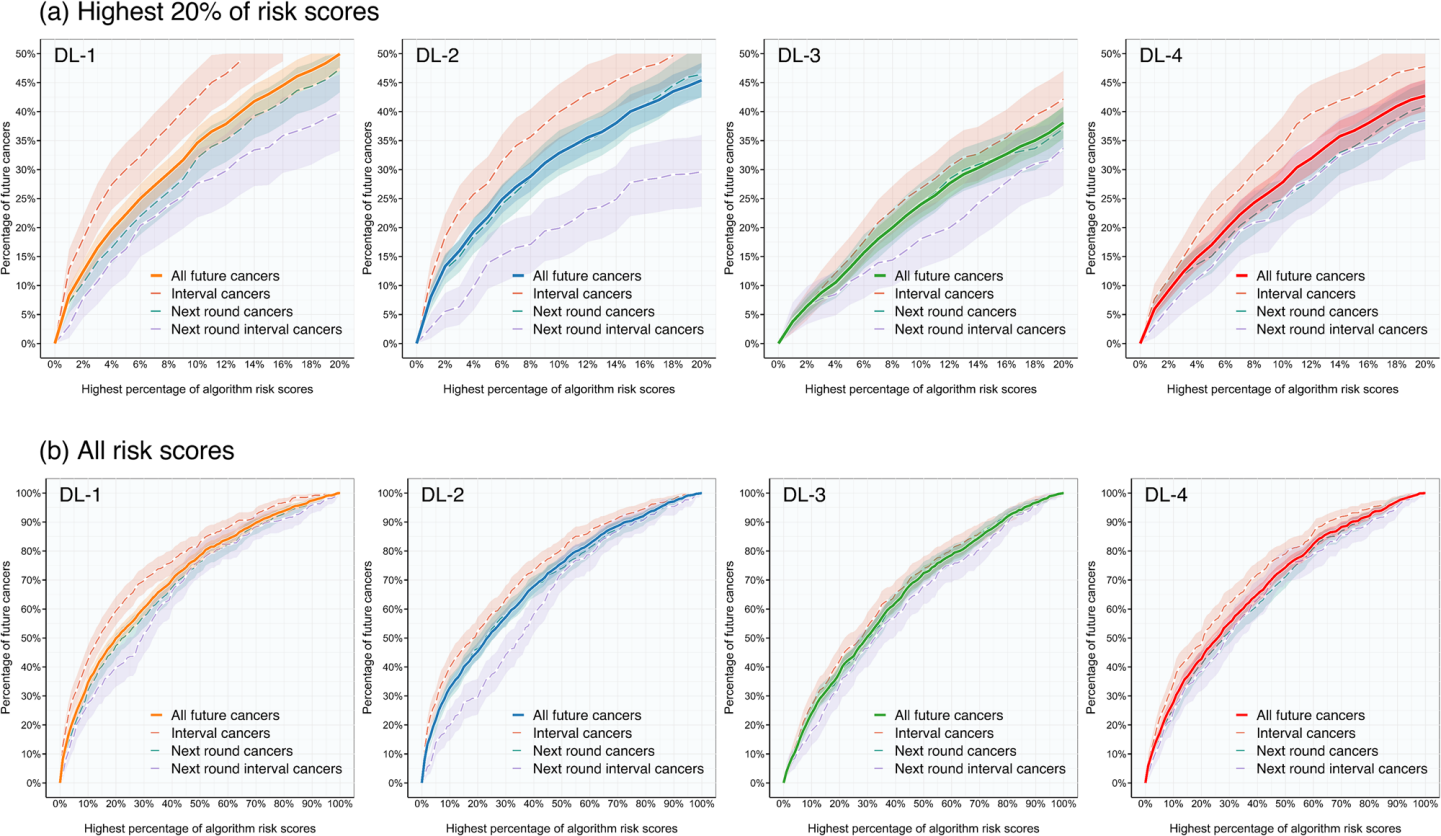
Gain charts relating algorithm risk scores to the proportions of correctly predicted future cancers. The relationship between operating thresholds set to positively identify the highest 1–100% of each algorithm’s respective risk scores, and proportions of correctly predicted future cancers and their subtypes (interval cancers, next round cancers, and next round interval cancers). Investigated percentages are shown in (**a**), with the full range provided in (**b**). Shaded regions represent 95% confidence intervals estimated via 2500 bootstrapped samples. DL Deep Learning algorithm.

Venn diagrams showed that algorithms identified partially overlapping subsets of future cancers; many true-positive cases were identified by only one algorithm, highlighting the significant heterogeneity in which individual cancers are flagged (Fig. S4 and S5). Results across a wider range of clinically relevant operating thresholds (highest 1.0%, 5.0%, 10.0%, and 20.0% of risk scores) are reported in Table 2, with corresponding gain charts in Fig. 5. Results are reported in Supplementary Information for the same analysis on Philips and GE mammograms alone (Tables S2 and S3).

**Table 2:** Algorithm correct predictions across clinically relevant operating thresholds

Operating threshold	DL-1	DL-2	DL-3	DL-4	*p*
**Highest 1.0% of scores**					
All future cancers***	8.3% [6.8–9.8%] (102/1,225)	8.2% [6.6–9.5%] (100/1,225)	3.8% [2.8–4.9%] (46/1,225)	5.9% [4.7–7.3%] (72/1,225)	< 0.001
ICs only***	12.9% [9.5–16.2%] (51/396)	11.4% [8.4–14.5%] (45/396)	3.5% [1.8–5.4%] (14/396)	7.6% [5.2–10.3%] (30/396)	< 0.001
NRCs only**	7.2% [5.3–9.2%] (44/613)	8.0% [5.9–10.0%] (49/613)	3.8% [2.4–5.4%] (23/613)	5.7% [4.0–7.8%] (35/613)	0.002
**NRICs only**	3.2% [0.9–5.4%] (7/216)	2.8% [0.9–5.2%] (6/216)	4.2% [1.8–7.1%] (9/216)	3.2% [0.9–5.6%] (7/216)	0.801
**Highest 4.0% of scores**					
**All future cancers**	19.7% [17.6–21.8%] (241/1,225)	19.3% [17.2–21.6%] (236/1,225)	10.4% [8.9–12.2%] (128/1,225)	14.9% [12.7–16.7%] (182/1,225)	< 0.001
**ICs only**	27.5% [23.3–31.8%] (109/396)	25.8% [21.7–30.2%] (102/396)	12.1% [8.9–15.5%] (48/396)	18.7% [14.7–22.4%] (74/396)	< 0.001
**NRCs only**	16.5% [13.6–19.5%] (101/613)	18.4% [15.6–21.6%] (113/613)	10.1% [8.0–12.7%] (62/613)	13.7% [11.0–16.2%] (84/613)	< 0.001
**NRICs only**	14.4% [9.8–19.2%] (31/216)	9.7% [5.9–14.0%] (21/216)	8.3% [4.9–12.6%] (18/216)	11.1% [7.1–15.8%] (24/216)	0.081
**Highest 5.0% of scores**					
All future cancers***	22.3% [19.9–24.6%] (273/1,225)	21.9% [19.5–24.2%] (268/1,225)	13.1% [11.1–14.9%] (160/1,225)	17.0% [14.9–19.2%] (208/1,225)	< 0.001
ICs only***	30.1% [25.4–34.6%] (119/396)	27.8% [23.3–32.2%] (110/396)	14.6% [11.3–18.3%] (58/396)	22.2% [18.3-–26.3%] (88/396)	< 0.001
NRCs only***	19.4% [16.4–22.6%] (119/613)	20.9% [17.7–24.1%] (128/613)	12.9% [10.1–15.6%] (79/613)	15.0% [12.1–17.9%] (92/613)	< 0.001
**NRICs only**	16.2% [11.5–21.3%] (35/216)	13.9% [9.4–18.7%] (30/216)	10.6% [6.7–14.8%] (23/216)	13.0% [8.8–17.9%] (28/216)	0.197
**Highest 10.0% of scores**					
All future cancers***	34.6% [31.9–37.3%] (424/1,225)	32.8% [30.2–35.6%] (402/1,225)	24.1% [21.6–26.5%] (295/1,225)	27.8% [25.2–30.4%] (340/1,225)	< 0.001
ICs only***	42.2% [37.4–47.4%] (167/396)	39.9% [35.2–44.8%] (158/396)	27.0% [22.6–31.2%] (107/396)	34.1% [29.8–39.1%] (135/396)	< 0.001
NRCs only***	32.1% [28.2–35.6%] (197/613)	32.8% [29.0–36.8%] (201/613)	24.3% [21.0–27.8%] (149/613)	24.8% [21.5–28.3%] (152/613)	< 0.001
NRICs only**	27.8% [21.8–33.5%] (60/216)	19.9% [14.7–25.5%] (43/216)	18.1% [13.0–23.4%] (39/216)	24.5% [18.9–30.3%] (53/216)	0.006
**Highest 14.0% of scores**					
**All future cancers**	41.8% [39.2–44.5%] (512/1,225)	38.0% [35.4–40.8%] (466/1,225)	30.4% [27.8–32.8%] (372/1,225)	35.7% [33.1–38.3%] (437/1,225)	< 0.001
**ICs only**	50.3% [45.2–55.0%] (199/396)	45.5% [40.2–50.0%] (180/396)	32.6% [28.2–37.3%] (129/396)	41.9% [37.0–46.4%] (166/396)	< 0.001
**NRCs only**	39.3% [35.6–43.1%] (241/613)	37.8% [34.2–41.8%] (232/613)	31.0% [27.4–34.6%] (190/613)	32.8% [29.1–36.4%] (201/613)	< 0.001
**NRICs only**	33.3% [27.4–40.1%] (72/216)	25.0% [19.5–30.9%] (54/216)	24.5% [18.6–30.3%] (53/216	32.4% [26.4–38.7%] (70/216)	0.004
**Highest 20.0% of scores**					
All future cancers***	50.0% [47.0–52.8%] (612/1225)	45.5% [42.6–48.4%] (557/1225)	38.0% [35.4–40.8%] (466/1225)	42.8% [40.0–45.5%] (524/1225)	< 0.001
ICs only***	59.3% [54.5–64.2%] (235/396)	52.5% [47.7–57.3%] (208/396)	41.9% [37.3–47.1%] (166/396)	47.7% [42.9–52.7%] (189/396)	< 0.001
NRCs only***	47.5% [43.3–51.5%] (291/613)	46.5% [42.4–50.5%] (285/613)	37.0% [33.2–40.9%] (227/613)	41.1% [37.0–44.9%] (252/613)	< 0.001
NRICs only*	39.8% [33.3–46.4%] (86/216)	29.6% [23.6–36.0%] (64/216)	33.8% [27.3–40.1%] (73/216)	38.4% [31.7–45.4%] (83/216)	0.010

The percentage of correctly predicted future cancers and each subtype at different clinically relevant operating thresholds. Percentages are accompanied by 95% confidence intervals estimated via 2,500 bootstrapped samples in square brackets, and the absolute number in parentheses. Cochran’s Q test was used to assess for heterogeneity in the four algorithm proportions. *p < 0.05, **p < 0.01, ***p < 0.001. *ICs* Interval Cancers, *NRCs* Next-Round screen-detected Cancers, *NRICs* Next-Round Interval Cancers.

## Discussion

This study demonstrates clear performance differences between current risk prediction algorithms, showing that discriminative metrics alone are insufficient to guide clinical interpretation of results. To the best of the authors’ knowledge, previous studies have not directly compared multiple named risk prediction algorithms using a UK screening cohort; this is also the first to validate the Google risk model. DL-1 consistently outperformed all other algorithms in predicting future cancers, with the highest discriminative ability (Figs. 3 and 4) and correct identification of nearly 30.0% of ICs within the highest 4.0% of risk scores (Fig. 5 (a)). AUCs were comparable to previous single algorithm validation studies^14,15,17,23,24^ and correctly predicted IC proportions were comparable to those found by Lång et al., who used DL risk scores to identify ICs that were visible on retrospective review^12^.

Increasing recall from the highest 4.0% of risk scores to 14.0% doubled the potential cancer yield, with the same algorithm performance rank order. No significant differences were found between correctly predicted NRICs proportions up to, but not after, the highest 5.0% of scores, suggesting equivalent predictive ability after 3-years at low threshold use (Table 2). Algorithms with high discriminative ability for ICs may be suitable to identify women for supplemental imaging, while those successfully identifying late-presenting cancers may provide more benefit in adjusting screening interval frequency. Using a subset of the data for which density was available, no differences in correctly predicted cancer characteristics were found between algorithms at 4.0% recall (Table 3). However, at 14.0% recall, DL-1 correctly predicted more cancers in dense breasts than all other algorithms (Table 4). Three of the four algorithms demonstrated consistent performance across the two mammography systems included in this study, indicating good generalizability confirmed by individual vendor analyses (Fig. 4; Tables S2 and S3). Furthermore, calibration and sensitivity analyses (Supplementary Information: Notes 3 and 4) support that algorithms predict clinically significant cancers, with the highest risk scores systematically assigned to mammograms preceding higher-grade invasive cancers; however, calibration was less applicable than in studies using predefined thresholds^25,26^, as discriminative ability was assessed at clinically defined recall rates rather than by applying fixed risk thresholds. All algorithms showed better predictive ability for cancers that presented shortly after screening (ICs) compared with those diagnosed later (NRCs and NRICs) (Figs. 2 and 4; Table 2). This suggests that algorithms may identify subtle signs of disease that are present but overlooked at screening, in addition to features associated with short-term risk. Venn diagrams also highlight that algorithms appear to identify different future cancers, with limited overlap (Fig. S4 and S5). This heterogeneity suggests that algorithm selection, or the use of ensembles, could influence which cancers are detected in practice.

**Table 3:** Characteristics of future cancer predictions within the highest 4.0% of algorithm risk scores (Philips, 2016 only)

	DL-1	DL-2	DL-3	DL-4	*p*
All future cancers*	20.6% [14.8–26.7%] (35/170)	20.6% [15.3–27.1%] (35/170)	13.5% [8.6–18.6%] (23/170)	15.3% [9.7–20.2%] (26/170)	0.019
**Cancer grade**					
**I**	8.7% [0.0–21.1%] (2/23)	13.0% [0.0–32.8%] (3/23)	4.3% [0.0–14.3%] (1/23)	4.3% [0.0–14.3%] (1/23)	0.629
**II**	23.0% [14.6–33.8%] (17/74)	25.7% [15.9–35.9%] (19/74)	17.6% [9.3–26.5%] (13/74)	17.6% [9.0–26.2%] (13/74)	0.192
**III**	21.1% [8.6–34.9%] (8/38)	13.2% [2.9–25.0%] (5/38)	18.4% [7.1–31.6%] (7/38)	21.1% [7.7–34.2%] (8/38)	0.653
DCIS*	21.4% [7.1–38.5%] (6/28)	17.9% [4.8–34.7%] (5/28)	0.0% [0.0–0.0%] (0/28)	10.7% [0.0–22.7%] (3/28)	0.024
**Unavailable**	28.6% [0.0–71.4%] (2/7)	42.9% [0.0–85.7%] (3/7)	28.6% [0.0–66.7%] (2/7)	14.3% [0.0–50.0%] (1/7)	0.392
**Molecular subtype**					
**Luminal A (ER**+**/PR**+**/HR2-)**	18.6% [9.9–28.9%] (13/70)	21.4% [12.7–32.4%] (15/70)	15.7% [7.6–24.6%] (11/70)	20.0% [10.6–29.6%] (14/70)	0.730
**Luminal B (ER**+**/PR-/HR2-)**	21.7% [5.3–40.0%] (5/23)	26.1% [8.7–45.0%] (6/23)	13.0% [0.0–28.3%] (3/23)	13.0% [0.0–26.8%] (3/23)	0.232
**Luminal B (ER**+**/PR**+**/-/HR2**+**)**	14.3% [0.0–50.0%] (1/7)	14.3% [0.0–50.0%] (1/7)	0.0% [0.0–0.0%] (0/7)	0.0% [0.0–0.0%] (0/7)	0.392
**HER2 positive (ER-/PR-/HR2**+**)**	12.5% [0.0–40.0%] (1/8)	12.5% [0.0–41.7%] (1/8)	25.0% [0.0–62.5%] (2/8)	12.5% [0.0–40.0%] (1/8)	0.801
**Triple negative (ER-/PR-/HR2-)**	11.1% [0.0–39.3%] (1/9)	11.1% [0.0–37.5%] (1/9)	22.2% [0.0–55.6%] (2/9)	0.0% [0.0–0.0%] (0/9)	0.494
Unavailable*	11.1% [0.0–39.3%] (1/53)	11.1% [0.0–37.5%] (1/53)	22.2% [0.0–55.6%] (2/53)	0.0% [0.0–0.0%] (0/53)	
**Volpara density grade**					
Non-dense (‘a’+‘b’)*	13.2% [6.8–21.1%] (12/91)	22.0% [14.4–31.4%] (20/91)	11.0% [4.8–17.5%] (10/91)	13.2% [6.4–20.0%] (12/91)	0.041
Dense (‘c’+‘d’)**	29.5% [20.0–39.4%] (23/78)	17.9% [10.1–26.7%] (14/78)	16.7% [9.0–25.0%] (13/78)	17.9% [9.0–26.1%] (14/78)	0.009
**Unavailable**	0.0% [0.0–0.0%] (0/1)	100.0% [100.0–100.0%] (1/1)	0.0% [0.0–0.0%] (0/1)	0.0% [0.0–0.0%] (0/1)	0.392

The percentage of future cancers within the highest 4.0% of algorithm risk scores and their respective characteristics. Results are for one site with mammograms from 2016 only, as this was the only data subset for which the Volpara density grade was available. This subset comprised 170 future cancers (including 52 ICs, 78 NRCs, and 40 NRICs), and 15,224 normal cases. Percentages are accompanied by 95% confidence intervals estimated via 2,500 bootstrapped samples in square brackets, and the absolute number in parentheses. Cochran’s Q test was used to assess for heterogeneity in the four algorithm proportions; no significant results were found following adjusted pairwise comparison. *p < 0.05, **p < 0.01, ***p < 0.001. *ICs* Interval Cancers, *NRCs* Next-Round screen-detected Cancers, *NRICs* Next-Round Interval Cancers.

**Table 4:** Characteristics of future cancer predictions within the highest 14.0% of algorithm risk scores (Philips, 2016 only)

	DL-1	DL-2	DL-3	DL-4	*p*
All future cancers***	40.6% [33.3–47.7%] (69/170)	38.2% [31.2–45.5%] (65/170)	24.1% [18.1–30.6%] (41/170)	32.9% [25.8–39.7%] (56/170)	< 0.001
**Cancer grade**					
**I**	21.7% [5.6–40.8%] (5/23)	30.4% [11.6–50.0%] (7/23)	26.1% [8.3–45.7%] (6/23)	26.1% [7.1–43.6%] (6/23)	0.857
II***	50.0% [38.9–61.3%] (37/74)	45.9% [34.7–57.7%] (34/74)	23.0% [13.8–32.9%] (17/74)	36.5% [25.7–47.2%] (27/74)	< 0.001
**III**	34.2% [19.0–50.0%] (13/38)	34.2% [18.9–50.0%] (13/38)	34.2% [18.9–50.0%] (13/38)	31.6% [16.7–46.3%] (12/38)	0.978
DCIS*	39.3% [20.7–58.2%] (11/28)	25.0% [9.4–41.9%] (7/28)	10.7% [0.0–24.1%] (3/28)	32.1% [16.0–52.2%] (9/28)	0.019
**Unavailable**	42.9% [0.0–85.7%] (3/7)	57.1% [16.7–100.0%] (4/7)	28.6% [0.0–66.7%] (2/7)	28.6% [0.0–71.4%] (2/7)	0.468
**Molecular subtype**					
**Luminal A (ER**+**/PR**+**/HR2-)**	38.6% [26.9–50.0%] (27/70)	41.4% [30.3–53.0%] (29/70)	25.7% [15.7–36.8%] (18/70)	31.4% [20.3–41.9%] (22/70)	0.051
**Luminal B (ER**+**/PR-/HR2-)**	43.5% [23.1–64.0%] (10/23)	34.8% [15.4–56.0%] (8/23)	26.1% [9.1–45.8%] (6/23)	39.1% [17.4–58.3%] (9/23)	0.471
**Luminal B (ER**+**/PR**+**/-/HR2**+**)**	42.9% [0.0–83.3%] (3/7)	42.9% [0.0–83.3%] (3/7)	0.0% [0.0–0.0%] (0/7)	14.3% [0.0–50.0%] (1/7)	0.145
**HER2 positive (ER-/PR-/HR2**+**)**	37.5% [0.0–75.0%] (3/8)	50.0% [10.0–87.5%] (4/8)	37.5% [0.0–75.0%] (3/8)	25.0% [0.0–62.5%] (2/8)	0.261
**Triple negative (ER-/PR-/HR2-)**	44.4% [10.0–80.0%] (4/9)	33.3% [0.0–66.7%] (3/9)	33.3% [0.0–66.7%] (3/9)	22.2% [0.0–55.6%] (2/9)	0.753
Unavailable*	44.4% [10.0-80.0%] (4/53)	33.3% [0.0–66.7%] (3/53)	33.3% [0.0–66.7%] (3/53)	22.2% [0.0–55.6%] (2/53)	0.005
**Volpara density grade**					
Non-dense (‘a’+‘b’)*	29.7% [20.3–39.3%] (27/91)	38.5% [28.7–48.8%] (35/91)	22.0% [13.6–30.7%] (20/91)	26.4% [16.8–35.5%] (24/91)	0.017
Dense (‘c’+‘d’)***	52.6% [41.9–63.9%] (41/78)	37.2% [26.5–47.7%] (29/78)	25.6% [16.7–35.9%] (20/78)	41.0% [30.7–51.3%] (32/78)	<0.001
**Unavailable**	100.0% [100.0–100.0%] (1/1)	100.0% [100.0–100.0%] (1/1)	100.0% [100.0–100.0%] (1/1)	0.0% [0.0–0.0%] (0/1)	0.392

The percentage of future cancers within the highest 14.0% of algorithm risk scores, and their respective characteristics. Results are for one site with mammograms from 2016 only, as this was the only data subset for which Volpara density grade was available. This subset comprised 170 future cancers (including 52 ICs, 78 NRCs, and 40 NRICs), and 15,224 normal cases. Percentages are accompanied by 95% confidence intervals estimated via 2,500 bootstrapped samples in square brackets, and the absolute number in parentheses. Cochran’s Q test was used to assess for heterogeneity in the four algorithm proportions. *p < 0.05, **p < 0.01, ***p < 0.001.

*ICs* Interval Cancers, *NRCs* Next-Round screen-detected Cancers, *NRICs* Next-Round Interval Cancers.

This study has several limitations. Non-standard examinations – those with implants or not comprising the standard four views (e.g., tessellating multiple images per view to visualize large volume breast tissue) – were excluded, as none of the included algorithms can currently process these cases. As this comprised a relatively high proportion of the initially collected dataset, this may not be wholly representative of real-world applications. Results may also not directly translate to other major mammography vendors in current screening practice (e.g., Hologic and Siemens). Ethnicity data were only available for <30% of women screened, so it was not possible to assess generalization across these groups or adjust comparisons. As a retrospective study, ground truth was limited to ICs that were undetected by mammographic screening and symptomatically presented between screening examinations. Therefore, this study likely underestimates correct prediction numbers, given the significant increase in cancer detection rates reported with risk-stratified supplemental imaging^11,20,21^.

A key strength of this study was the use of full cohort data from two independent NHSBSP sites, representative of routine triennial screening on two different mammography systems. The results support previous validation studies and maintain that clinically useful breast cancer risk information can be obtained from routine screening mammograms in the absence of additional risk information. By reporting failure outcomes and directly comparing commercial and academic algorithms, this study provides policymakers with clearer performance benchmarks relating to national recall rates. These results highlight the importance of considering site capacity and the mammography system when selecting algorithms.

It is imperative that future work reports technical reliability of algorithms and robustness to changes in the mammography system or associated software, with accompanying failure analysis. Prospective trials should evaluate the real-world clinical benefit and cost-effectiveness of the best-performing algorithms for risk-stratified screening with adjusted frequency and supplemental imaging.

## Methods

### Study data

This retrospective validation study used NHSBSP full-field digital screening mammograms and associated National Breast Screening System (NBSS) data from screening cohorts at two independent sites in the same geographic region for one triennial screening round (April 1st, 2014, to March 31st, 2017). The NHSBSP invites women from age 50-to-70-years for mammographic screening every three-years; women aged 71 and older may self-refer to continue screening. Women at high-risk of breast cancer (e.g., BRCA1 carriers) are invited for more frequent screening from an earlier age, and may be offered supplemental MRI^27^. Mammograms from the first site (Cambridge and Huntingdon) were obtained using Philips Healthcare Sectra MicroDose L30 systems, and the second site (Norfolk and Norwich) using GE HealthCare Senographe Essential systems.

These data were obtained from the Cambridge Cohort Mammography East Anglia Digital Imaging Archive (‘CC-MEDIA’) database, which comprises pseudonymized NHBSP data. Mammography systems and implants were identified using DICOM headers. CC-MEDIA has ethical approval in which informed consent is waived (Health Research Authority Research Ethics Committee 25/LO/0220, Health Research Authority Confidentiality Advisory Group 20/CAG/0009, Public Health England Research Advisory Committee BSPRAC_090). Clinical trial number: not applicable.

### Study population

The chosen population had at least five years of follow-up. Normal case ground truth was no cancer diagnosis within five-years of the baseline negative screening mammogram. Cancer cases were histopathologically confirmed, and grade and molecular subtype were obtained where possible. Each was categorised as an IC, NRC, or NRIC, with ICs and NRICs presenting symptomatically between screening rounds, and NRCs detected at the screening round subsequent to the baseline mammogram. When multifocal or bilateral, the cancer with the worst prognostic stage was used, according to the American Joint Committee on Cancer (AJCC) staging system.

For this study, mammograms were excluded if they were baseline screen-detected cancer, part of annual high-risk screening, included implants, or could not be processed by all algorithms. Examinations were also excluded if they did not exactly comprise one of each standard mammographic view (craniocaudal and mediolateral oblique for both lateralities), as these cases cannot currently be processed by the included algorithms. The time interval in days from baseline screening mammogram to cancer diagnosis was obtained from NBSS for all cancers.

### Validated algorithms

Four risk prediction algorithms were compared: Mirai (version 0.5.0), iCAD ProFound AI Risk (version 2.0; iCAD, Inc.^28^), Transpara Risk (version 1.0.1; ScreenPoint Medical^29^), and the Google risk model (version 2.0). Each is reported under an alias in this paper: DL-1, DL-2, DL-3, or DL-4, masking the identity of each algorithm, as is our agreement with each company.

Mirai is a widely-validated academic algorithm developed by Yala et al. using approximately 210,000 mammograms obtained on Hologic systems at Massachusetts General Hospital in Boston, MA^30^, and is available under the MIT License^31^. iCAD ProFound AI Risk^13^ is CE marked and Health Canada Licensed. Transpara Risk^12^ is not yet commercially available and was provided for research purposes. Both were trained using national screening data obtained on multiple mammography systems; further development details are described by Gastounioti et al. for iCAD ProFound AI Risk^23^ and Lauritzen et al. for Transpara Risk^24^. The Google risk model was developed by Google Health using the dataset and global model described by McKinney et al.^32^ iCAD has a development and commercialization agreement with Google Health, enabling integration of Google’s AI technology into their ProFound Breast Health Suite for 2D Mammography, for use worldwide.

Algorithms processed negative screening mammograms to produce scores estimating the likelihood of breast cancer developing within three-years of that examination, within a local Linux-based environment with four CUDA-enabled GPUs on a single machine (Nvidia RTX 6000 Ada Generation, 48 GB memory). Reprocessing was attempted for cases that initially failed for each algorithm, and failure analysis was discussed with algorithm companies. At no point was this environment accessible to companies, including study data and algorithms, to prevent bias and ensure consistent processing.

### Algorithm assessment

The AUC for each algorithm was estimated for predicting any future cancer within five-years of each baseline negative screening mammogram, to assess overall discriminative performance. Statistically significant differences were assessed with DeLong’s test, using 10,000 bootstrap samples and Holm-Bonferroni adjustment for multiple comparisons. No formal a priori power calculation was performed. The sample size in this study exceeded that in previously published evaluations of these algorithms^10,12,13,17,23,24,30,32^ and was therefore considered adequate to detect clinically meaningful differences in AUC.

Subgroup analyses were undertaken to assess and compare AUCs for the two mammography systems. Future cancers were also split into two groups to investigate the extent to which detection plays a role: those that were more likely to have been present at the baseline mammogram (ICs alone), and those that were not likely to be present, and developed after screening (NRCs and NRICs combined). Absolute calibration over the study period was assessed for probabilistic algorithms, with relative calibration evaluated for score-based algorithms; full results are provided in the Supplementary Information (Note 2).

The proportion of correctly predicted future cancers at all operating thresholds was determined for each algorithm and used to construct gain charts, with 2,500 bootstrapped samples to estimate 95% confidence intervals. Corresponding sub-analyses were undertaken for future cancer groups. Correct predictions at select clinically relevant thresholds were obtained to contextualise results, including use of the highest 4.0% of risk scores, corresponding to NHSBSP ‘acceptable’ incident screen recall rate^33^, and the highest 14.0%, corresponding to the upper limit of a range of reported North American recall rates (5.0–14.0%)^34^.

Logistic regression was used with a subset (one year of Philips data) for which Volpara density grade was available, to assess the association between true positive predictions for each algorithm and cancer characteristics (time-to-diagnosis, grade, molecular subtype, density, and patient age). Volpara density grades of ‘a’ and ‘b’ were considered non-dense; ‘c’ and ‘d’ were considered dense. Predictions for a wider range of thresholds (highest 1.0%, 5.0%, 10.0%, and 20.0% of risk scores) were then investigated. Cochran’s Q test was used to assess for heterogeneity in the proportions of correctly predicted cancers across the four algorithms. If heterogeneity was found, pairwise post-hoc McNemar’s tests were performed with Holm-Bonferroni correction for multiple comparisons. Minimum sample sizes necessary to find differences between paired algorithm predictions at each threshold were estimated post-hoc using McNemar’s test, assuming 80% power and a two-sided alpha of 0.05. All comparisons were sufficiently powered, except for those comparing the proportion of future cancers correctly predicted by DL-1-and-2 on all data (Table 2), due to similar true positive prediction rates with low discordance.

All analyses were undertaken in R (version 4.4.3; R Foundation for Statistical Computing) by two authors, and findings were considered statistically significant when *p* < 0.05.

## Supplementary information

Supplementary information

## Data Availability

Study data from the UK NHS Breast Screening Programme were pseudonymised. Access is restricted, and the data cannot be publicly shared. However, researchers may request access through the relevant NHS data governance and research ethics procedures, subject to review and appropriate data sharing agreements. Requests should be made to the corresponding author and will be considered on a case-by-case basis.

## Code availability

The deep learning algorithms evaluated in this study were provided for research purposes by their respective commercial companies and cannot be shared by the authors. Researchers wishing to access these algorithms should contact the respective companies directly. Mirai has been made available by Yala et al. on GitHub: https://github.com/yala/Mirai. Analysis code for this study has been made available on GitHub: https://github.com/RandomForestJosh/breast_cancer_risk_prediction.
