# Supplementary material for: Performance of breast cancer risk prediction algorithms across mammography systems in the UK screening programme
Source: NPJ Digit Med. 2026 Mar 8;9:330. doi: 10.1038/s41746-026-02507-7 (PMC13096106; doi:10.1038/s41746-026-02507-7)
Supplement: Supplementary file 1 — Supplementary information [file 41746_2026_2507_MOESM1_ESM.pdf]

Supplementary Information

Supplementary Note 1: Deep learning (DL) algorithm processing failure reasons and countermeasures

For DL-1:

- 1,121 cases initially failed to process
- Following discussion with developers, mammograms from women younger than 40 or older than 80 years of age were excluded, as calibration of this algorithm to absolute risk is not valid outside this age range
- 711 / 1,121 of the failed cases were >80 years of age; 0 were younger than 40 years
- Of the remaining 410 cases, risk scores were successfully obtained for 368 cases following a second round of processing, with unclear reason for their initial failure
- Of the remaining 43, 36 were found to include supplementary views (e.g. XCCL) in addition to the four standard mammographic views; scores were successfully obtained for these cases following removal of the additional views
- Two cases of the remaining cases were found to not contain the patient age DICOM tag, and were successfully processed once this was added
- One of the remaining cases contained the correct patient age in the DICOM tags for 2/4 images, but the incorrect age in the other 2/4 images; the true patient age was checked and the incorrect DICOM tags corrected, leading to successful processing
- For the remaining two cases, one view of the first case could not be displayed in a DICOM viewer and was thought to be corrupt; an error code (‘Corrupt JPEG data: bad Huffman code’) was obtained for the second case

For DL-2:

- 16 cases initially failed to process
- Risk scores were successfully obtained for 12 of these cases following a second round of processing, with unclear reason for their initial failure
- Two cases were found to not contain the patient age DICOM tag, and were successfully processed once this was added; this was a confirmed failure reason with the developer
- Of the remaining two cases, one view of the first case could not be displayed in a DICOM viewer and was thought to be corrupt; an error code (‘Corrupt JPEG data: bad Huffman code’) was obtained for the second case

For DL-3:

- Two cases initially failed to process
- One case was successfully processed when reattempted, with unclear reason for initial failure
- One case failed to process (error code: ‘Corrupt JPEG data: bad Huffman code’)

For DL-4:

- 337 cases initially failed to process
- Seven cases did not contain spot compression, but were found to falsely contain the spot compression DICOM tag in one examination view, with this failure reason confirmed by the developer; all seven cases were successfully processed following removal of the spot compression tag
- Logs for the remaining 330 cases are currently under further investigation at the time of submission

Time (seconds)	DL-1	DL-2	DL-3	DL-4
Philip example case				
Median time [IQR]	15 [15, 16]	44 [43, 45]	10 [10, 10]	79 [77, 81]
1	18	45	10	84
2	14	45	10	77
3	15	45	10	77
4	15	41	10	76
5	15	43	10	79
6	15	46	10	82
7	16	41	10	78
8	16	45	10	79
9	19	44	10	79
10	15	42	10	76
11	13	44	10	84
12	15	43	10	76
13	13	44	10	82
14	15	43	10	75
15	19	43	10	79
GE example case				
Median time [IQR]	12 [12,13]	35 [34,37]	8 [8 8]	67 [66, 68]
1	12	34	8	69
2	12	35	8	63
3	14	35	8	66
4	12	37	8	68
5	12	33	8	63
6	11	35	8	66
7	13	36	8	68
8	14	36	8	68
9	12	37	8	68
10	11	34	8	67
11	15	38	8	73
12	12	33	8	65
13	13	37	8	70
14	11	34	8	66
15	12	32	9	65

Table S1: Example processing times for each algorithm on identical mammograms, using one GPU

All numbers are time in seconds; median time is accompanied by the interquartile range. GPUs were Nvidia RTX 6000 Ada Generation (48 GB memory).

DL = Deep Learning algorithm.

Supplementary Note 2: Assessment of algorithm calibration

Calibration refers to the agreement between predicted risk estimates and observed cancer incidence. Well-calibrated models ensure that a women assigned a given percentage risk of breast cancer experience cancer at approximately that rate.

We assessed calibration by plotting the predicted versus observed cancer incidence for each algorithm using all study data, followed by vendor-specific and grade-specific (Table S4-1) calibration curves

Importantly, only DL-1 and DL-3 were trained to produce calibrated probability estimates and could therefore be directly evaluated against a y = x reference line representing 'perfect' calibration. In contrast, DL-2 and DL-4 output relative risk scores rather than absolute probabilities; their evaluation was therefore limited to relative calibration between mammography vendors, examining whether predicted risk rankings corresponded to observed outcome frequencies in a consistent manner.

DL-1 achieved excellent calibration with predicted risk closely matching observed cancer incidence for all study data, and across both mammography vendors (Figure S4-1: (a)). For Philips mammograms, slight overestimation was observed in the moderate and highest risk deciles, with slight underestimation for GE mammograms within the highest risk decile.

DL-3 achieved good calibration at lower risk deciles (Figure S4-1: (b)) but with systematic overestimation for moderate and the highest risk deciles for Philips mammograms, and severe underestimation for the highest risk decile of GE mammograms, indicating poorer calibration consistency across vendors.

For DL-2 and DL-4 (Figures S4-1: (c) (d)), the relationship between mean predicted risk and observed cancer incidence was comparable between vendors, with substantial overlap of estimates across most risk deciles. Observed cancer incidence increased with predicted risk comparably for both algorithms, though DL-4 showed greater dispersion within the highest risk decile.

Grade-specific calibration plots support that these algorithms predict clinically significant cancers (Figure S4-2). The highest risk scores for all algorithms were systematically assigned to mammograms preceding Grade II interval cancers, followed by Grade III, then DCIS and Grade I interval cancers. This indicates that algorithms not only discriminate between cancer and non-cancer cases but appropriately assign higher risks to mammograms preceding more clinically significant invasive cancers.

Interval cancer type	Total	Median age at negative screening	Median time to symptomatic presentation
Grade I and DCIS	73	57 [51, 63]	643 [459, 828]
Grade II	162	59 [52, 66]	642 [415, 869]
Grade III	115	59 [53, 65]	698 [470, 927]

Table S2: Interval cancer subgroups by histological grade

Median times are accompanied by interquartile range in brackets.

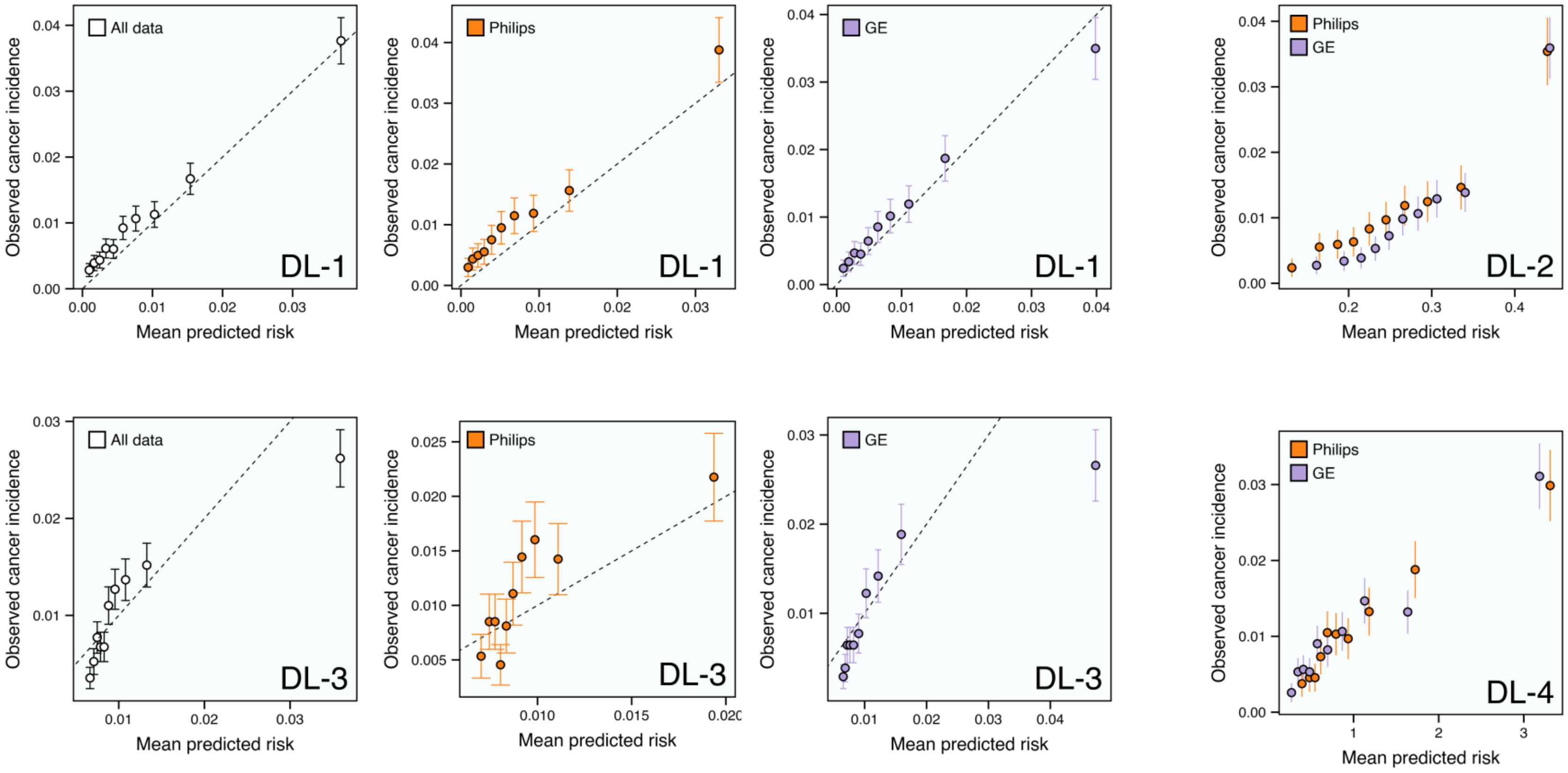

Figure S1: Overall calibration by mammography vendor

Calibration plots showing observed interval cancer incidence versus mean predicted risk across deciles of predicted risk, stratified by mammography vendor (Philips: orange; GE: purple). Each point represents one risk decile. For DL-1 (a) and DL-3 (b), which output risk probability estimates, the diagonal dashed line represents ‘perfect’ calibration (observed = predicted). DL-2 (c) and DL-4 (d) were not trained to produce probability estimates, so only relative calibration between risk deciles and mammography vendors can be assessed.

DL = Deep Learning algorithm.

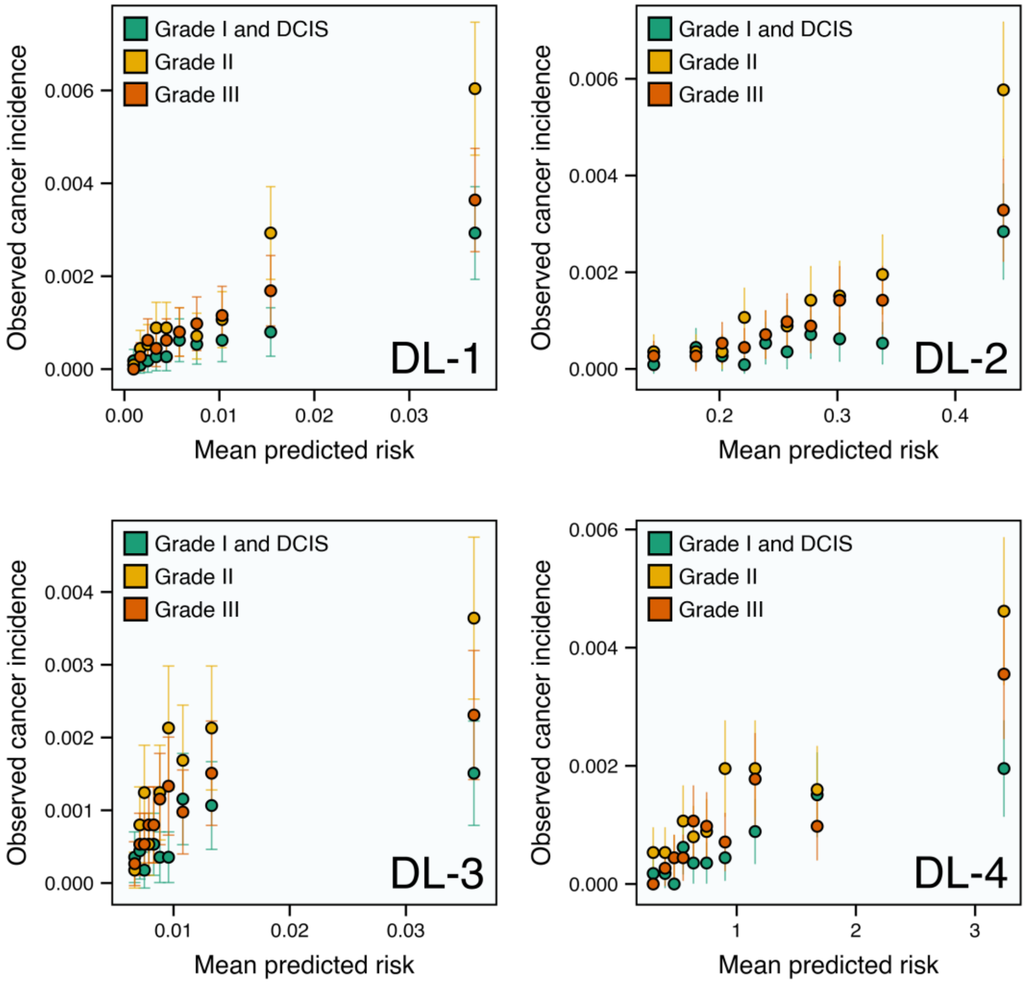

Figure S2: Interval cancer incidence by histological grade across risk deciles
Observed interval cancer incidence by histological grade across deciles of predicted risk, stratified into subgroups: Grade I and DCIS (green), Grade II (yellow), Grade III (red). Each point represents one risk decile.

DL = Deep Learning algorithm.

Supplementary Note 3: AUC comparisons for separate mammography systems

This Material reports the estimated area under the receiver operating characteristic curves (AUCs) for each algorithm on separate mammography systems, extending the main text's Results section to include 95% confidence intervals.

Restricted to Philips mammograms (Figure 3: (b)), AUC performance patterns remained consistent with the overall data. Discriminating between normal cases and 'early' future cancers, DL-1 (0.78 [95% CI: 0.74–0.81]) outperformed DL-2 (0.74 [95% CI: 0.70–0.78]) (p=0.02), DL-3 (0.63 [95% CI: 0.58–0.67]; p<0.001), and DL-4 (0.73 [95% CI: 0.70–0.77]) (p=0.020). Again, DL-2-and-4 were comparable (p=0.77) and both outperformed DL-3 (p<0.001). Comparing these results to the 'later' future cancers subgroup, patterns remained consistent to the overall data. Statistical decreases were found for DL-1 (0.78 to 0.68 [95% CI: 0.65–0.71]; p<0.001), DL-2 (0.74 to 0.67 [95% CI: 0.64–0.70]; p=0.003), and DL-4 (0.73 to 0.65 [95% CI: 0.63–0.68]; p<0.001). DL-3 (0.63 to 0.61 [95% CI: 0.59–0.64] remained comparable (p=0.65). Within this subgroup, DL-1's AUC remained highest (0.69); it did not differ from DL-2 (0.67; p=0.47) and trended against DL-4 (0.66; p=0.058). All outperformed DL-3 (0.61; DL-1-and-2: p<0.001; DL-4: p=0.026).

Restricted to GE mammograms (Figure 3: (c)), performance differences were smaller. Discriminating between normal cases and 'early' future cancers, DL-1 (0.76 [95% CI: 0.73–0.80]) was superior to DL-3 (0.70 [95% CI: 0.67-0.74]; p<0.001) and DL-4 (0.72 [95% CI: 0.68-0.75]; p=0.002), but comparable with DL-2 (0.75 [95% CI: 0.71-0.78]; p=0.36), with no other pairwise IC comparisons significant. Comparing these results to the 'later' future cancers subgroup, statistical decreases were seen for DL-1 (0.76 to 0.70 [95% CI: 0.68–0.73]; p=0.004), DL-2 (0.75 to 0.69 [95% CI: 0.67–0.72]; p=0.011), and DL-4 (0.72 to 0.67 [95% CI: 0.64–0.69]; p=0.016), but not for DL-3 (0.70 to 0.67 [95% CI: 0.65–0.69]; p=0.12). Within this subgroup, DL-1 (0.70 [95% CI: 0.68–0.73]) and DL-2 (0.69 [95% CI: 0.67–0.72]) were comparable (p=0.54); both outperformed DL-3 (0.67 [95% CI: 0.65–0.69]; p=0.011 for DL-1, p=0.079 for DL-2) and DL-4 (0.67 [95% CI: 0.65–0.69]; DL-1: p=0.003; DL-2: p=0.044). DL-3 and DL-4 were comparable (p=0.65).

DL = Deep Learning algorithm. ICs = Interval Cancers. NRCs = Next-Round screen-detected Cancers. NRICs = Next-Round Interval Cancers.

Supplementary Note 4: Interval cancer subtype sensitivity analyses

It is clinically important to assess the phenotype of correct predictions made by deep learning (DL) algorithms – i.e. if there is preferential prediction of more aggressive cancers, as these carry the poorest prognosis and benefit most from earlier diagnosis and intervention.

The sensitivity of each algorithm to more aggressive cancers was therefore assessed by splitting interval cancers into three subgroups – less aggressive (Grade I and DCIS) interval cancers, Grade II interval cancers, and Grade III interval cancers (Table S3-1). Figure S3-1 presents the proportion of interval cancers that presented symptomatically and were correctly predicted in two-month intervals following negative screening, stratified by subgroup at operating thresholds corresponding to recall of the highest 4% (Figure S3-1: (a)) and 14% (Figure S3-1: (b)) of risk scores.

At 4% recall, all algorithms demonstrated an early peak in the proportions of correctly predicted interval cancers within the first 6 months following negative screening, likely representing identification of cancers with detectable features already present at screening. DL-1 and DL-2 predicted the highest proportion of interval cancers within the first 12 months; Grade I and DCIS constituted the largest proportion, followed by Grade III cancers for DL-1 and Grade II cancers for DL-2. In contrast, DL-3 and DL-4 identified very few Grade I cancers and DCIS within the first 12 months, also predicting fewer Grade II and III cancers compared with DL-1 and DL-2.

Increasing recall to 14% resulted in higher sustained correct prediction proportion across the 36-month screening round for all algorithms. DL-1 achieved the highest proportions. Grade-specific patterns were consistent with the 4% threshold, though relative contributions varied more over time at the higher recall rate – particularly Grade III interval cancers for DL-1, which increased to over 60% in the first 12 months.

For all algorithms, correct predictions proportions stabilized at lower levels for later-presenting Grade I, DCIS and Grade III interval cancers, but were broadly consistent for Grade II interval cancers across the 36-month screening round. This temporal pattern supports the notion that these algorithms detect subtle signs of disease that are present but overlooked at screening in addition to features associated with short-term risk. These findings also demonstrate that these algorithms show preferential prediction of moderate-to-high grade invasive interval cancers in the immediate post-screening period, with varying grade-sensitivity patterns suggesting that algorithm selection could influence which cancer phenotypes are found if implemented into risk-stratified screening programmes.

Interval cancer type	Total	Median age at negative screening	Median time to symptomatic presentation
Grade I and DCIS	73	57 [51, 63]	643 [459, 828]
Grade II	162	59 [52, 66]	642 [415, 869]
Grade III	115	59 [53, 65]	698 [470, 927]

Table S3-1: Interval cancer subgroups by histological grade

Median times are accompanied by interquartile range in brackets.

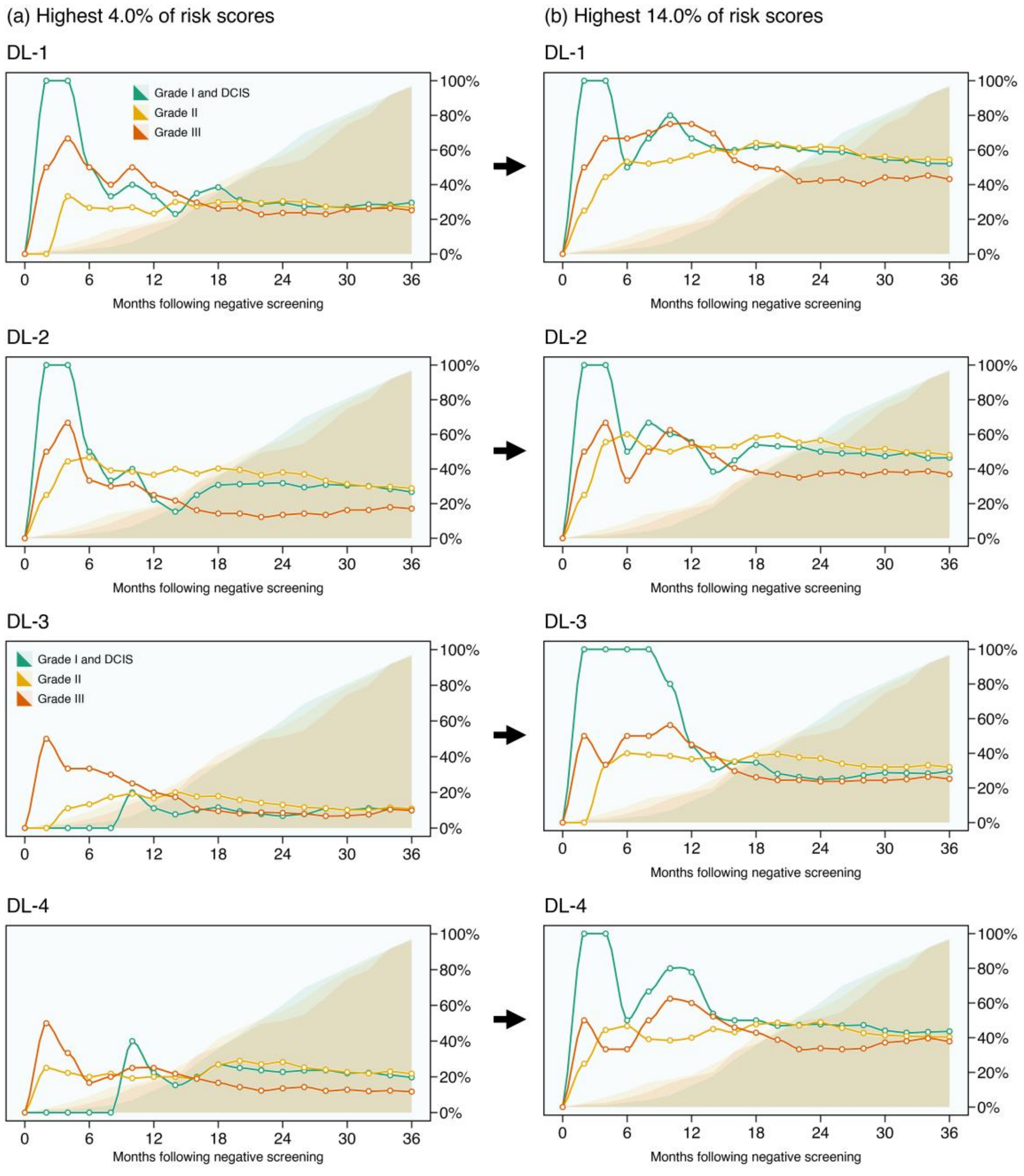

Figure S3: Prediction sensitivity to interval cancer histological grade over time

The proportion of interval cancers that symptomatically presented and were correctly predicted by each algorithm within two-month intervals, stratified by histological grade, at risk thresholds corresponding to 4.0% (a) and 14.0% (b) recall rates. Points represent different interval cancer subgroups: Grade I and DCIS (green), Grade II (yellow), Grade III (red). Shaded regions represent cumulative cancer incidence for each subgroup over 36 months following negative screening.

DL = Deep Learning algorithm.

Operating threshold	DL-1	DL-2	DL-3	DL-4	*p*
Highest 1.0% of scores					
All future cancers***	8.3% [6.1-10.5%] (46/1,225)	8.6% [6.4-11.0%] (48/1,225)	3.1% [1.7-4.7%] (17/1,225)	6.5% [4.5-8.6%] (36/1,225)	<0.001
ICs only***	13.2% [8.5-18.1%] (23/396)	12.1% [7.6-16.8%] (21/396)	3.4% [1.1-6.5%] (6/396)	8.6% [4.7-13.0%] (15/396)	<0.001
NRCs only*	7.0% [4.2-9.8%] (20/613)	8.0% [4.9-11.4%] (23/613)	3.1% [1.3-5.3%] (9/613)	5.9% [3.4-8.9%] (17/613)	0.042
NRICs only	3.2% [0.0-7.7%] (3/216)	4.2% [0.9-8.3%] (4/216)	2.1% [0.0-6.5%] (2/216)	4.2% [1.0-8.5%] (4/216)	0.786
Highest 5.0% of scores					
All future cancers***	23.6% [20.1-27.0%] (131/1,225)	21.6% [18.3-25.1%] (120/1,225)	13.1% [10.4-16.1%] (73/1,225)	17.1% [14.1-20.3%] (95/1,225)	<0.001
ICs only***	29.3% [22.8-36.3%] (51/396)	28.7% [22.1-35.6%] (50/396)	14.4% [9.1-19.8%] (25/396)	23.6% [17.4-30.0%] (41/396)	<0.001
NRCs only**	20.6% [15.9-25.0%] (59/613)	19.5% [14.9-24.2%] (56/613)	12.5% [9.1-16.5%] (36/613)	14.6% [10.7-18.7%] (42/613)	0.003
NRICs only	22.1% [14.1-30.6%] (21/216)	14.7% [8.4-23.0%] (14/216)	12.6% [6.1-19.4%] (12/216)	12.6% [6.4-19.4%] (12/216)	0.070
Highest 10.0% of scores					
All future cancers***	34.7% [30.7-38.4%] (193/1,225)	31.7% [27.7-35.4%] (176/1,225)	18.7% [15.7-22.0%] (104/1,225)	26.8% [23.1-30.3%] (149/1,225)	<0.001
ICs only***	46.0% [38.7-52.9%] (80/396)	39.1% [31.7-46.1%] (68/396)	22.4% [16.2-29.0%] (39/396)	33.9% [26.9-41.1%] (59/396)	<0.001
NRCs only***	30.3% [25.2-35.6%] (87/613)	30.0% [24.7-35.3%] (86/613)	16.7% [12.9-21.4%] (48/613)	23.0% [18.0-27.7%] (66/613)	<0.001
NRICs only	27.4% [18.5-36.3%] (26/216)	23.2% [15.1-32.1%] (22/216)	17.9% [10.4-26.2%] (17/216)	25.3% [16.7-34.1%] (24/216)	0.184
Highest 20.0% of scores					
All future cancers***	48.6% [44.3-52.7%] (270/1,225)	44.6% [40.2-48.6%] (248/1,225)	31.5% [27.8-35.3%] (175/1,225)	43.3% [39.2-47.3%] (241/1,225)	<0.001
ICs only***	61.5% [54.3-68.6%] (107/396)	50.6% [43.1-57.7%] (88/396)	31.0% [24.5-38.2%] (54/396)	48.3% [40.6-55.9%] (84/396)	<0.001
NRCs only***	43.6% [37.9-49.1%] (125/613)	44.6% [38.8-50.3%] (128/613)	32.8% [27.6-38.3%] (94/613)	40.8% [34.9-46.2%] (117/613)	<0.001
NRICs only*	40.0% [30.2-50.0%] (38/216)	33.7% [23.5-43.0%] (32/216)	28.4% [19.3-38.4%] (27/216)	42.1% [32.2-52.8%] (40/216)	0.027

Table S3: Algorithm correct predictions across clinically relevant operating thresholds, Philips mammograms only

The percentage of correctly predicted future cancers and each subtype at different clinically relevant operating thresholds. Percentages are accompanied by 95% confidence intervals estimated via 2,500 bootstrapped samples in square brackets, and the absolute number in parentheses. Cochran's Q test was used to assess for heterogeneity in the four algorithm proportions. $*p<0.05$, $**p<0.01$, $***p<0.001$.

DL = Deep Learning algorithm. ICs = Interval Cancers. NRCs = Next-Round screen-detected Cancers. NRICs = Next-Round Interval Cancers.

Operating threshold	DL-1	DL-2	DL-3	DL-4	*p*
Highest 1.0% of scores					
All future cancers***	8.1% [6.3-10.3%] (54/1,225)	7.6% [5.7-9.6%] (51/1,225)	3.7% [2.3-5.2%] (25/1,225)	5.5% [4.0-7.3%] (37/1,225)	<0.001
ICs only***	11.3% [7.2-15.5%] (25/396)	10.8% [6.8-15.1%] (24/396)	3.6% [1.4-6.2%] (8/396)	7.2% [4.1-10.9%] (16/396)	<0.001
NRCs only	7.7% [5.1-10.9%] (25/613)	7.4% [4.6-10.4%] (24/613)	4.3% [2.2-6.6%] (14/613)	5.5% [3.3-8.2%] (18/613)	0.118
NRICs only	3.3% [0.8-6.9%] (4/216)	2.5% [0.0-5.4%] (3/216)	2.5% [0.0-5.5%] (3/216)	2.5% [0.0-5.6%] (3/216)	0.968
Highest 5.0% of scores					
All future cancers***	21.7% [18.6-24.8%] (145/1,225)	21.7% [18.7-25.0%] (145/1,225)	14.6% [12.0-17.4%] (98/1,225)	17.3% [14.5-20.2%] (116/1,225)	<0.001
ICs only***	31.1% [25.2-37.0%] (69/396)	26.6% [20.7-32.6%] (59/396)	15.8% [11.1-20.6%] (35/396)	21.2% [16.0-26.6%] (47/396)	<0.001
NRCs only**	18.1% [13.8-22.3%] (59/613)	21.8% [17.6-26.5%] (71/613)	14.7% [10.9-18.5%] (48/613)	16.3% [12.3-20.4%] (53/613)	0.009
NRICs only	14.0% [8.1-20.3%] (17/216)	12.4% [6.7-18.5%] (15/216)	12.4% [6.7-18.3%] (15/216)	13.2% [7.2-20.0%] (16/216)	0.956
Highest 10.0% of scores					
All future cancers***	32.9% [29.3-36.3%] (220/1,225)	33.8% [30.4-37.3%] (226/1,225)	25.3% [22.2-28.4%] (169/1,225)	29.1% [25.6-32.3%] (195/1,225)	<0.001
ICs only***	40.5% [34.2-46.9%] (90/396)	40.5% [34.0-47.0%] (90/396)	27.9% [21.8-33.6%] (62/396)	34.7% [28.6-41.0%] (77/396)	<0.001
NRCs only**	30.7% [25.5-35.8%] (100/613)	35.3% [30.3-40.5%] (115/613)	26.1% [21.6-31.0%] (85/613)	27.0% [22.1-31.7%] (88/613)	0.001
NRICs only	24.8% [17.4-33.0%] (30/216)	17.4% [10.6-24.6%] (21/216)	18.2% [11.6-25.2%] (22/216)	24.8% [16.3-32.1%] (30/216)	0.141
Highest 20.0% of scores					
All future cancers***	50.4% [46.7-54.2%] (337/1,225)	46.9% [43.3-50.7%] (314/1,225)	43.0% [39.5-46.7%] (288/1,225)	42.6% [38.9-46.2%] (285/1,225)	<0.001
ICs only**	56.8% [50.0-63.3%] (126/396)	53.2% [47.1-59.5%] (118/396)	45.5% [39.1-52.0%] (101/396)	49.1% [42.1-55.7%] (109/396)	0.002
NRCs only**	50.0% [44.9-55.5%] (163/613)	49.4% [44.0-54.7%] (161/613)	45.7% [40.1-51.1%] (149/613)	40.8% [35.3-46.2%] (133/613)	0.002
NRICs only	39.7% [30.7-48.7%] (48/216)	28.9% [21.0-37.4%] (35/216)	31.4% [23.6-40.1%] (38/216)	35.5% [26.9-44.3%] (43/216)	0.072

Table S4: Algorithm correct predictions across clinically relevant operating thresholds, GE mammograms only

The percentage of correctly predicted future cancers and each subtype at different clinically relevant operating thresholds. Percentages are accompanied by 95% confidence intervals estimated via 2,500 bootstrapped samples in square brackets, and the absolute number in parentheses. Cochran's Q test was used to assess for heterogeneity in the four algorithm proportions. *$p<0.05$, **$p<0.01$, ***$p<0.001$.

DL = Deep Learning algorithm. ICs = Interval Cancers. NRCs = Next-Round screen-detected Cancers. NRICs = Next-Round Interval Cancers.

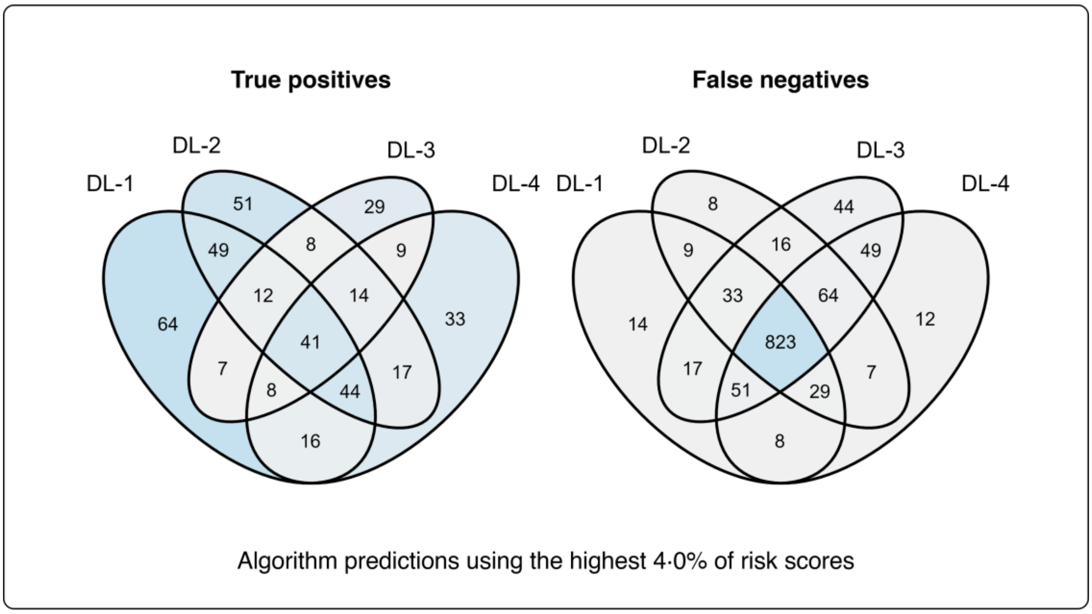

Figure S4: Venn diagrams for the number of true positive and false negative predictions made by each algorithm at an operating threshold corresponding to a recall rate of 4.0%

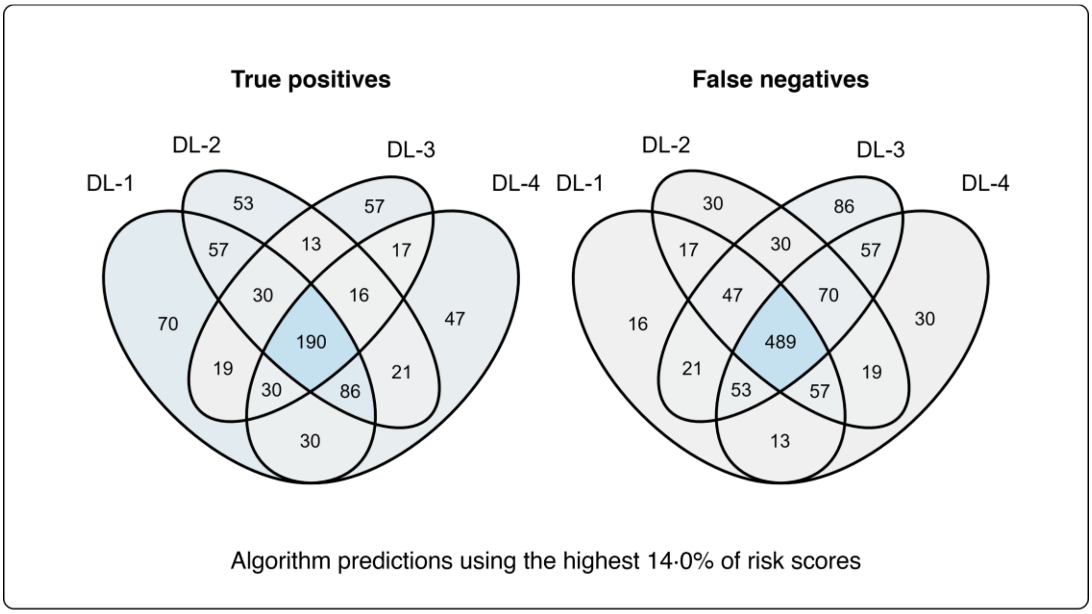

Figure S5: Venn diagrams for the number of true positive and false negative predictions made by each algorithm at an operating threshold corresponding to a recall rate of 14.0%
